# Supplementary material for: Frameshifting at collided ribosomes is modulated by elongation factor eEF3 and by integrated stress response regulators Gcn1 and Gcn20
Source: RNA. 2022 Mar;28(3):320–39. doi: 10.1261/rna.078964.121 (PMC8848926; doi:10.1261/rna.078964.121)
Supplement: Supplemental Material [file supp_28_3_320__DC1.html]

Frameshifting at collided ribosomes is modulated by elongation factor eEF3 and by integrated stress response regulators Gcn1 and Gcn20 — Supplemental Material 

# Frameshifting at collided ribosomes is modulated by elongation factor eEF3 and by integrated stress response regulators Gcn1 and Gcn20

## Supplemental Material

- Supplemental\_Fig\_S1.pdf
- Supplemental\_Fig\_S2.pdf
- Supplemental\_Fig\_S3.pdf
- Supplemental\_Fig\_S4.pdf
- Supplemental\_Fig\_S5.pdf
- Supplemental\_Fig\_S6.pdf
- Supplemental\_Figure\_Legends.docx
- Supplemental\_Table\_S1.docx
- Supplemental\_Table\_S2.xlsx
- Supplemental\_Table\_S3.xlsx
- Supplemental\_Table\_S4.xlsx
