# Supplementary figures and images for: Frameshifting at collided ribosomes is modulated by elongation factor eEF3 and by integrated stress response regulators Gcn1 and Gcn20
Source: RNA. 2022 Mar;28(3):320–39. doi: 10.1261/rna.078964.121 (PMC8848926; doi:10.1261/rna.078964.121)

SF 1

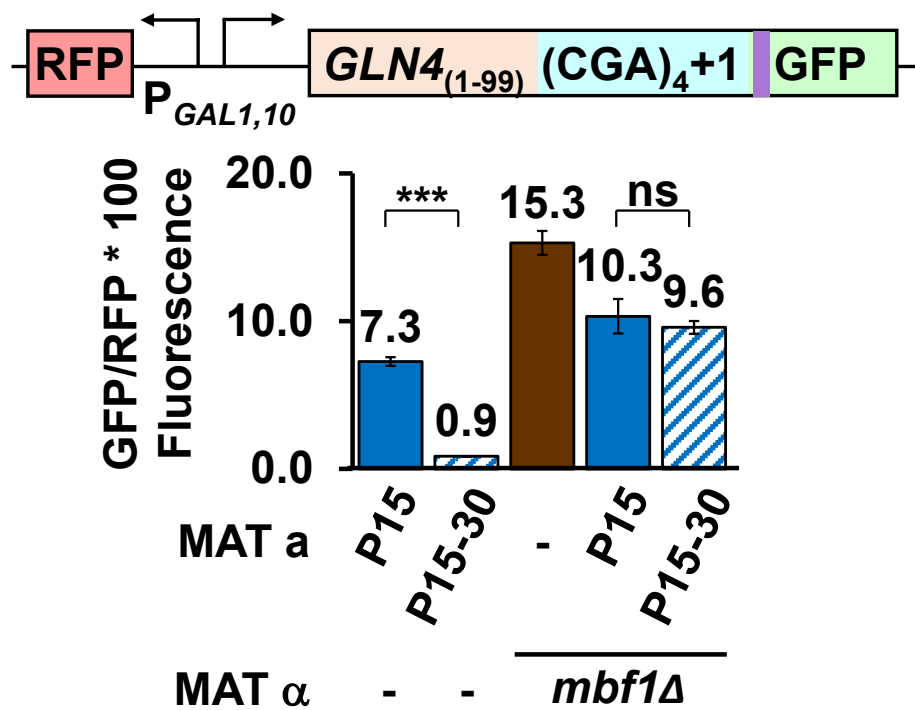

Supplement: Supplemental Material [file supp_078964.121_Supplemental_Fig_S1.pdf]

SF 3

A

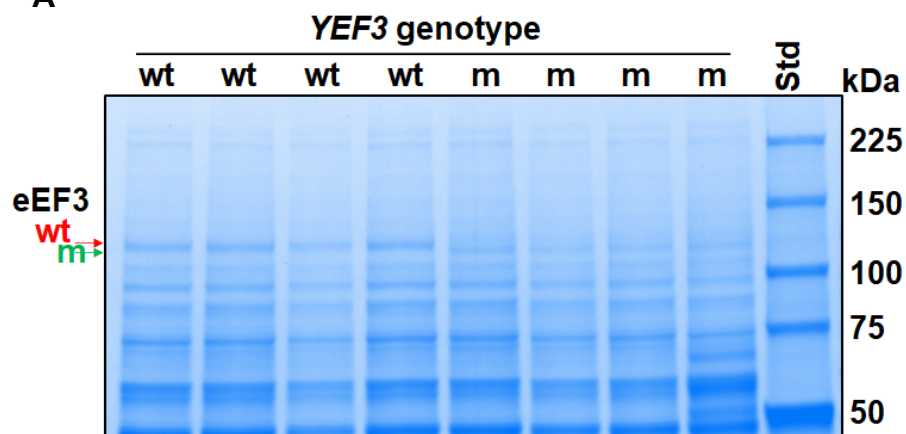

B

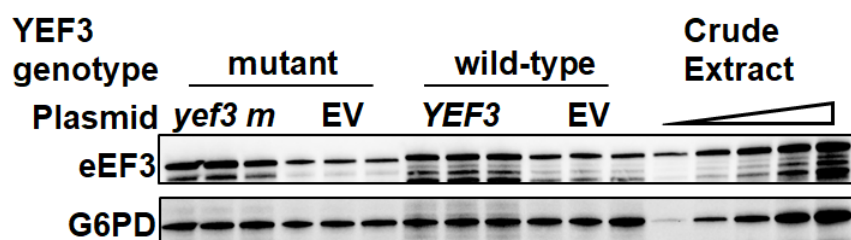

C

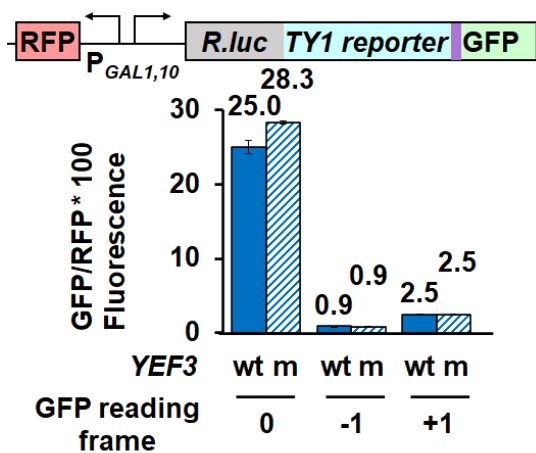

D

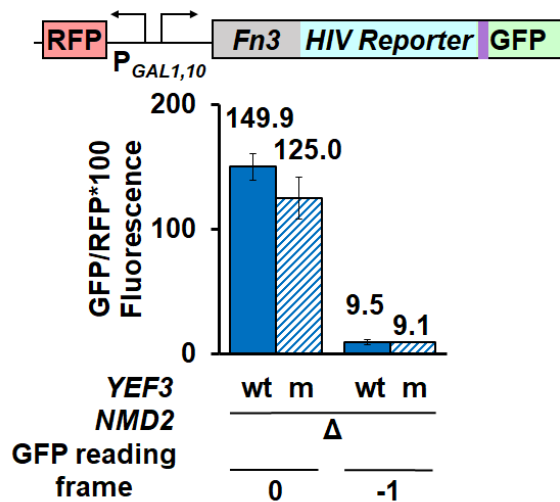

Supplement: Supplemental Material [file supp_078964.121_Supplemental_Fig_S3.pdf]

SF 4

A

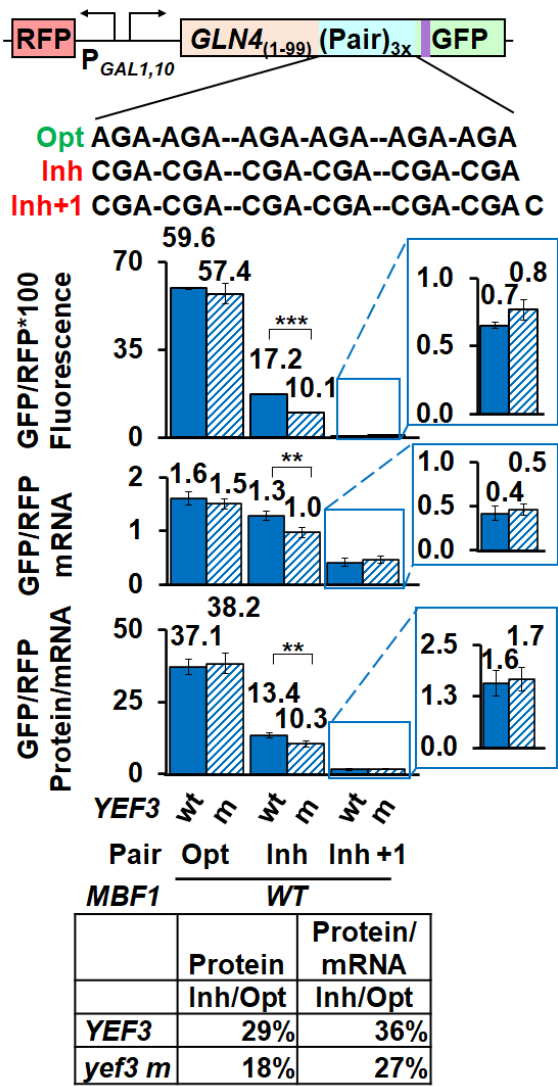

B

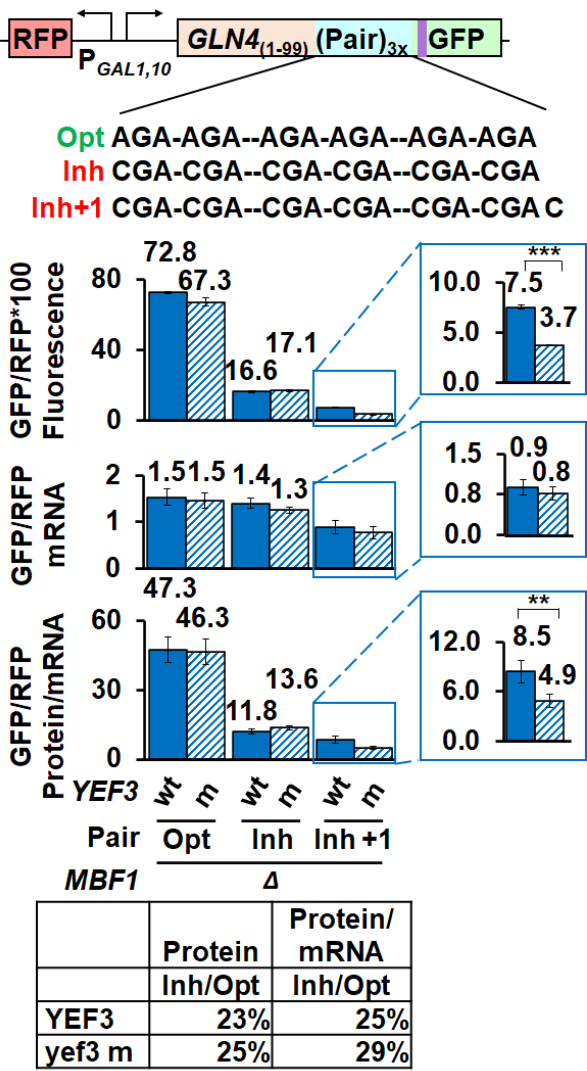

Supplement: Supplemental Material [file supp_078964.121_Supplemental_Fig_S4.pdf]

# SF 5

**A**

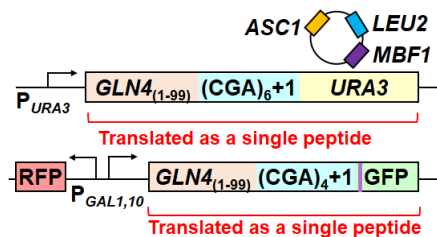

**B**

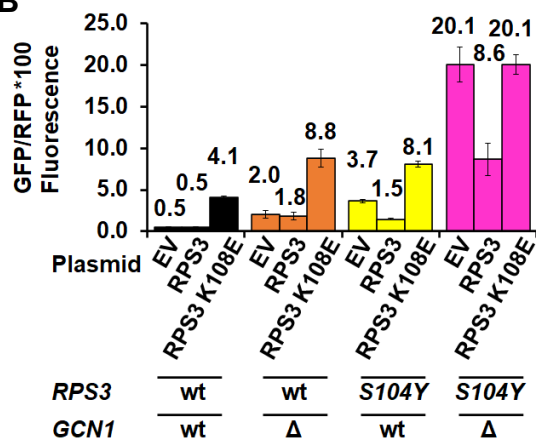

**C**

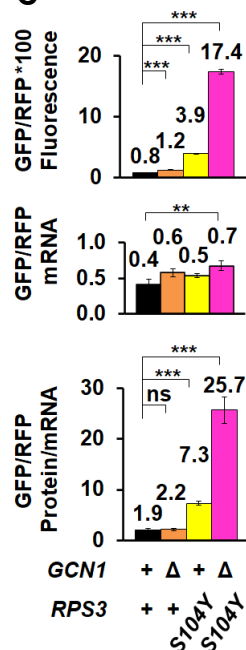

**D**

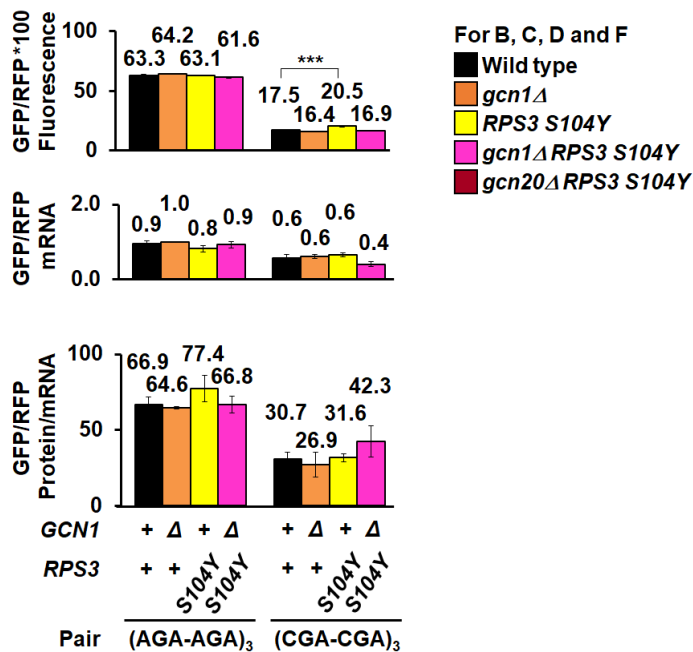

**E**

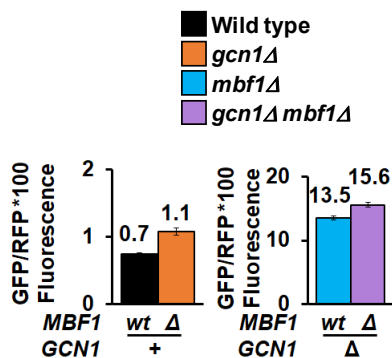

**F**

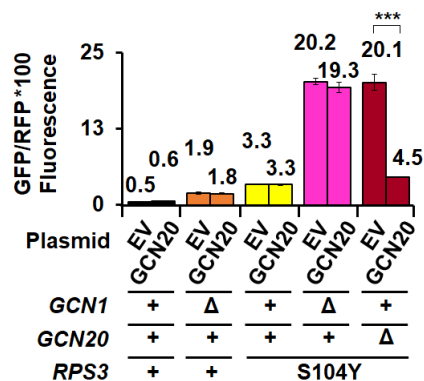

Supplement: Supplemental Material [file supp_078964.121_Supplemental_Fig_S5.pdf]

SF 6

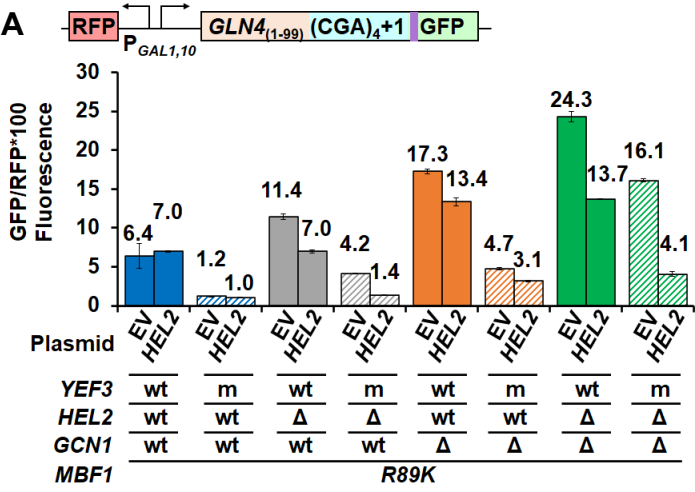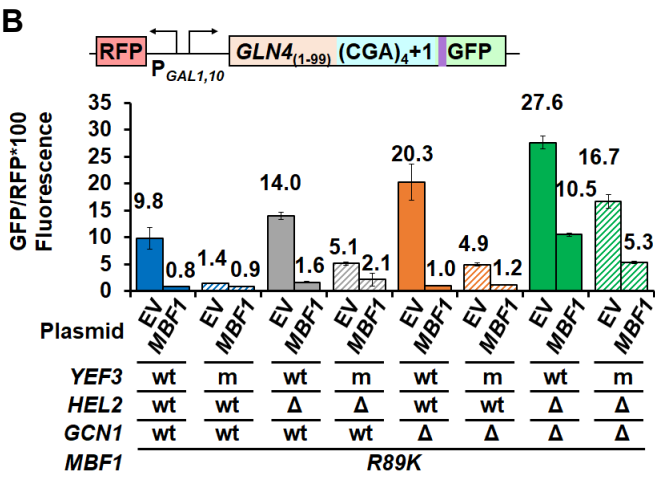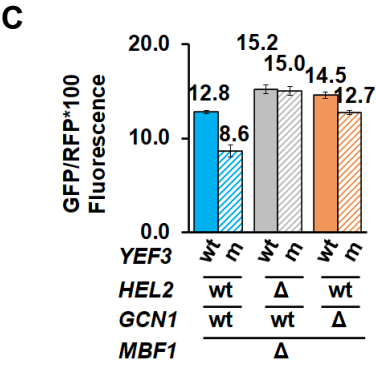

Supplement: Supplemental Material [file supp_078964.121_Supplemental_Fig_S6.pdf]
