# Supplementary material for: Frameshifting at collided ribosomes is modulated by elongation factor eEF3 and by integrated stress response regulators Gcn1 and Gcn20
Source: RNA. 2022 Mar;28(3):320–39. doi: 10.1261/rna.078964.121 (PMC8848926; doi:10.1261/rna.078964.121)
Supplement: Supplemental Material [file supp_078964.121_Supplemental_Fig_S2.pdf]

SF 2

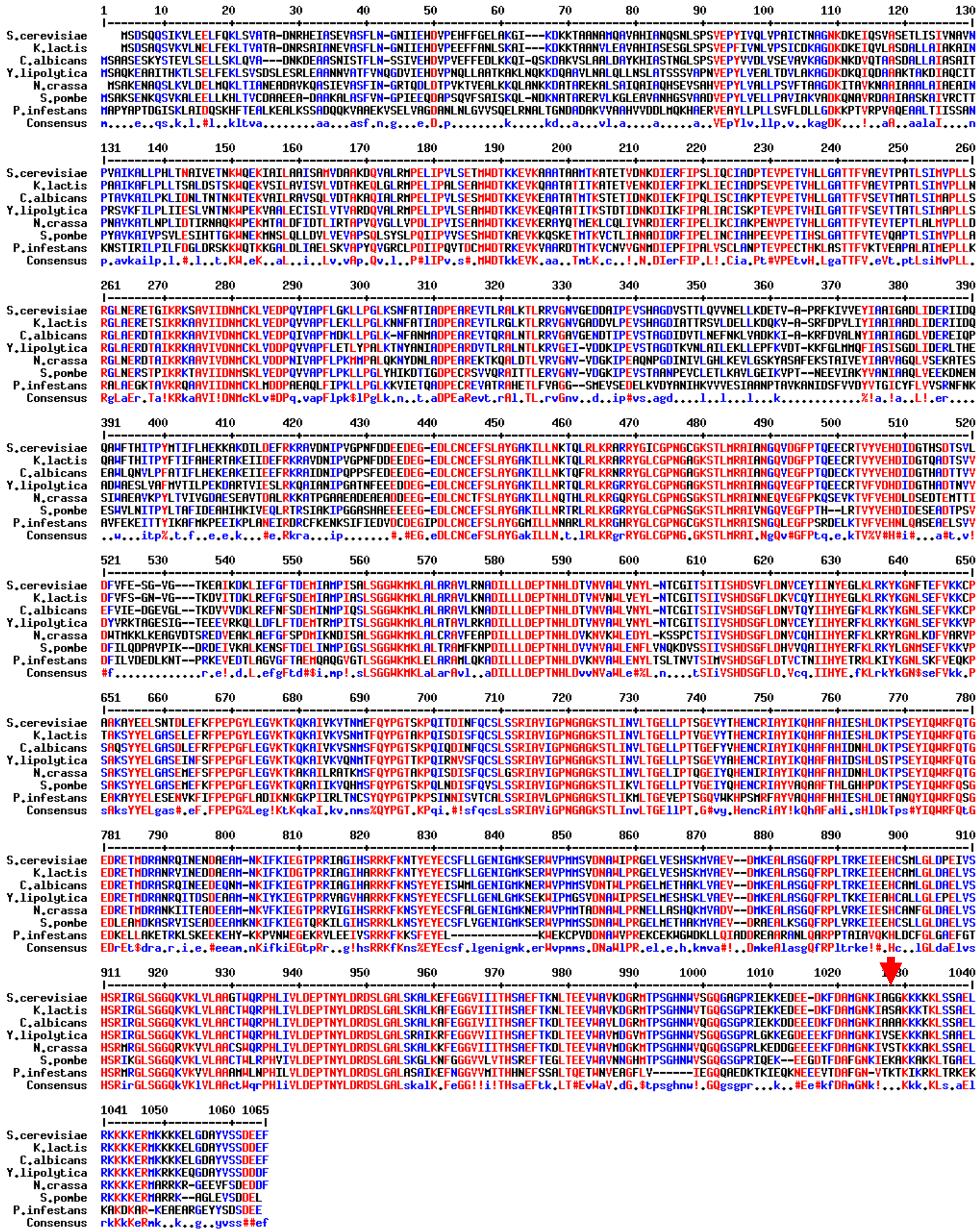
S.cerevisiae
K.lactis
C.albicans
Y.lipolytica
N.crassa
S.pombe
P.infestans
Consensus
