# Supplementary material for: Frameshifting at collided ribosomes is modulated by elongation factor eEF3 and by integrated stress response regulators Gcn1 and Gcn20
Source: RNA. 2022 Mar;28(3):320–39. doi: 10.1261/rna.078964.121 (PMC8848926; doi:10.1261/rna.078964.121)
Supplement: Supplemental Material [file supp_078964.121_Supplemental_Figure_Legends.docx]

**Supplemental Figure S1. Suppressor P15-30 is recessive**. Expression of frameshifted GFP/RFP is significantly lower in the haploid *MAT****a*** P15-30 compared to that in *MAT****a*** P15, but expression of the frameshifted GFP/RFP is nearly identical for diploids of both *MAT****a*** P15 and *MAT****a*** P15-30 mated with *MATα* *mbf1Δ* (*MAT****a***/*MATα* *mbf1Δ*/*mbf1-R89K YEF3/YEF3 and MAT****a***/*MATα* *mbf1Δ*/*mbf1 R89K YEF3/yef3 G1007V K1009fs*)*.*

**Supplemental Figure S2. Amino acid sequence alignment of eEF3 from six *Ascomycete* fungi across several different clades and a verified eEF3 from the Chromista *P. infestans* (Mateyak et al. 2018) using MultAlin** ([http://multalin.toulouse.inra.fr/multalin/](http://multalin.toulouse.inra.fr.ezpminer.urmc.rochester.edu/multalin/)) (Corpet 1988). The color text represents the level of consensus for each residue (Blue: 50-90%, Red: >90%). The red arrow indicates the location of the *yef3-fs1009* mutations.

**Supplemental Figure S3. The *yef3-fs1009* mutation alters eEF3 amounts, which can be restored by additional copies of the mutant form, and does not affect *TY1* or *HIV1* frameshifting.** (A) Strains with the *yef3-fs1009* mutation have reduced levels and altered migration of eEF3 protein compared to otherwise isogenic strains with wild type *YEF3*. Crude extracts from the indicated strains were subjected to SDS-PAGE and Coomassie-staining. (B) eEF3 levels in the *yef3-fs1009* mutant expressing *yef3-fs1009* on a *CEN* plasmid exceed the levels of eEF3 in a *YEF3* wild type strain with an empty vector. Crude lysates separated by SDS-PAGE were subjected to Western analysis with anti-eEF3 and anti-glucose-6-phosphate-dehydrogenase (G6PD) antibodies. (C) The *yef3-fs1009* mutant does not affect *TY1* programmed frameshifting. GFP/RFP levels from GFP constructs bearing the *TY1* frameshifting sequence in the indicated reading frames were examined in strains with *YEF3* wild type and *yef3-fs1009*. (D) The *yef3-fs1009* mutant does not affect *HIV1* programmed frameshifting. GFP/RFP levels from GFP constructs bearing the *HIV1* frameshifting sequence in the indicated reading frames were examined in strains with *YEF3* wild type and *yef3-fs1009*.

**Supplemental Figure S4.** **CGA codon pairs are inhibitory in *yef3-fs1009* mutants with either wild type *MBF1* (A)or no *MBF1 (mbf1Δ* (B).** GFP/RFP protein (fluorescence), mRNA and protein/mRNA were examined from the inhibitory (CGA-CGA)_3_ and optimal (AGA-AGA)_3_ in-frame reporters as well as the frameshifted (CGA-CGA)_3_+1 reporter. As expected, the GFP/RFP from the frameshifting reporter (CGA-CGA)_3_+1 was near background levels with wild type MBF1(A) (see Materials and Methods), but was much greater in *mbf1Δ* strains (B). Frameshifted GFP/RFP was significantly lower in the *yef3-fs1009 mbf1Δ* mutant compared to the *YEF3* *mbf1Δ *mutant.

**Supplemental Figure S5.** **Effects of** ***GCN1*, *RPS3* and *GCN20* on frameshifting depend upon the mutations in these genes, are not mediated through effects on in-frame expression and do not depend upon a functional copy of *GCN2*.** (A) Schematic of the parental strain YJYW290 used for selection of mutants that promote frameshifting (Wang et al. 2018). (B) Exogenous expression of wild type *RPS3* or frameshifting mutant *RPS3-K108E* results in expected effects on frameshifted GFP/RFP. Wild type *RPS3* suppresses the frameshifting in the *RPS3-S104Y* single mutant and in the *gcn1Δ* *RPS3-S104Y* double mutant but has no effect on frameshifting in the *gcn1Δ* single mutant. *RPS3-K108N* results in increased frameshifting in the wild type, *gcn1Δ*, and *RPS3-S104Y* mutants, but has no detectable effect in the *gcn1Δ* *RPS3-S104Y* double mutant. (C) The increase in frameshifting in the *gcn1Δ* *RPS3 S104Y* double mutant is not due to effects on mRNA levels. The *gcn1Δ* *RPS3-S104Y* double mutant significantly increases expression of both GFP/RFP protein and protein per mRNA but has only small effects on the mRNA for the frameshifted reporter. (D) Expression of the in-frame reporters is not affected by the *gcn1Δ* mutation*,* *RPS3-S104Y* mutation, or the *gcn1Δ* *RPS3-S104Y* double mutations. (E) Deletion of *GCN1* does not result in a substantial increase in frameshifting in strains lacking the *MBF1* gene. Results from strains identical to those in Fig. 5C, but grown in rich media (YP raffinose galactose). Frameshifting in *gcn1Δ* strain is low due to growth conditions. (F) Expression of *GCN20* results in reduced frameshifting in the *gcn20Δ* *RPS3-S104Y* double mutant but has no effect on frameshifting in the *gcn1Δ RPS3-S104Y* double mutant.

**Supplemental Figure S6.** Exogenous expression of *HEL2* and *MBF1* modulate frameshifting. (A) Expression of *HEL2* complements *hel2Δ* and *hel2Δ gcn1Δ *in both* YEF3* and *yef3-fs1009* strains, resulting in a reduction in frameshifted GFP/RFP to levels at or below those in the corresponding *HEL2* strain. Moreover, expression of *HEL2* also results in a reduction in frameshifted GFP/RFP in *gcn1Δ* mutants. (B) Expression of *MBF1* from a multicopy plasmid resulted in a reduction in frameshifted GFP/RFP in all strains, suppressing frameshifting to near background levels in the parent and single mutant strains. However, MBF1 expression only partially suppressed frameshifting in the *hel2Δ gcn1Δ* mutants. (C) The *yef3-fs1009* mutation suppresses frameshifting inefficiently in strains lacking *MBF1*.
