# Supplementary material for: Frameshifting at collided ribosomes is modulated by elongation factor eEF3 and by integrated stress response regulators Gcn1 and Gcn20
Source: RNA. 2022 Mar;28(3):320–39. doi: 10.1261/rna.078964.121 (PMC8848926; doi:10.1261/rna.078964.121)
Supplement: Supplemental Material [file supp_078964.121_Supplemental_Table_S1.docx]

Supplemental Table S1

| Sequence ID | YJYW ID | Mutant ID | Gene and mutation | GFP/RFP |
| --- | --- | --- | --- | --- |
| YW50 | YJYW10 | Q5 | *gcn1(61*)* | 0.69 |
| YW52 | YJYW12 | Q74 | *gcn1(471fs)* | 4.80 |
| YW53 | YJYW13 | Q94 | *gcn1(26*) **** | 2.63 |
| YW54 | YJYW14 | Q96 | *gcn1(1138*)* | 5.31 |
| YW55 | YJYW15 | Q110 | *gcn1(1033*)* | 3.54 |
| YW57 | YJYW31 | Q3 | *hel2 and gcn1(1001*)* | 7.20 |
| YW58 | YJYW32 | Q6 | *gcn1(403*)* | 1.11 |
| YW63 | YJYW17 | YJYW3183 | Parent | 0.01 |
